# Association Between Raja Isteri Pengiran Anak Saleha Appendicitis (RIPASA) Scoring and Pathological Severity of Acute Appendicitis: A Cross-Sectional Study

**DOI:** 10.7759/cureus.56166

**Published:** 2024-03-14

**Authors:** Arshad Koroth, Shiraz Basheer, Muhamed Fawas Abdul Rasheed, Azif Ali Usman, Arjun Sadanandan

**Affiliations:** 1 Department of General Surgery, Kasturba Medical College, Manipal, Manipal, IND; 2 Department of General Surgery, Muslim Educational Society (MES) Academy of Medical Sciences, Perinthalmanna, IND

**Keywords:** ripasa scoring system, negative appendicectomy, appendicular perforation, appendicular abscess, appendicitis

## Abstract

Background and objectives

Appendicitis is a frequent cause of abdominal pain. Because of the limited availability of imaging services in many medical centers and an urge to reduce the substantial number of unnecessary appendectomies, several clinical diagnostic tools have been constructed. A novel diagnostic tool, referred to as the Raja Isteri Pengiran Anak Saleha Appendicitis (RIPASA) score, has been created to assist in identifying acute appendicitis (AA) in Asian nations. The study aimed to assess the correlation between RIPASA scores and the severity of appendicitis as determined by pathological examination.

Materials and methods

The study was a prospective observational investigation undertaken in the Department of General Surgery at Muslim education society (MES) Medical College Perinthalmanna over 12 months. The study included all patients who had been diagnosed with AA and underwent appendectomy, provided they satisfied both the inclusion and exclusion criteria. An analysis was conducted on a convenience sample of 225 individuals using a prestructured proforma. The RIPASA scores were estimated before their surgery, taking into account their age, gender, symptoms, physical examination findings, and laboratory findings. These scores were subsequently contrasted with the histopathological results obtained after the appendectomy. The individuals were categorized into three groups according to their RIPASA scores. The lower-score category, scores between 4 and 7. The intermediate-score category consists of scores ranging from 7.5 to 11.5, while the higher-score category includes scores of 12 and above. These scores are correlated with the histopathology report (HPR) to determine the presence of appendicitis, perforated appendix, appendicular abscess, or the absence of pathology observed.

Results

The study population comprised of 137 (60.9%) males and 88 (39.1%) females. Among these, 177 individuals (78.7%) were younger than 40 years, while 48 individuals (21.3%) were older than 40 years. Out of 225 cases, 146 cases were AA (64.9%), 27 (12%) appendicular abscess cases, and 41 (18.2%) appendicular perforation cases. The normal appendix was noted in 11 out of 225 cases in the low-score group. The association between the histopathological report and RIPASA score was found to be statistically highly significant (p=0.000). In the low-score group, there were 14 cases of appendicitis (53.8%), one case of appendicular abscess (3.8%), a total of 11 cases without pathology observed (42.3%), and no reported instances of appendicular perforation. In the intermediate-score category, there were 121 cases of appendicitis (89.6%), 12 cases of appendicular abscess (8.9%), 2 cases of appendicular perforation (1.5%), and no reported cases in the non-pathology category. Among the high-score category, there were 11 cases of appendicitis (17.2%), 14 cases of appendicular abscess (21.9%), 39 cases of appendicular perforation (60.9%), and no reported instances of negative appendectomy.

Conclusion

The study has shown that the RIPASA scoring system had a high diagnostic efficacy in identifying AA. This scoring system is an effective, dependable, cost-effective, noninvasive, reproducible, and safe diagnostic technique that does not require additional expenses or concerns.

## Introduction

Acute appendicitis (AA), which refers to the inflammation of the appendix, is a frequent surgical emergency that poses a major threat to the general population. It has an average lifetime occurrence rate of 8.6% in men and 6.7% in women. The highest frequency is typically observed between the ages of 20 and 30 [1]. The diagnosis of this condition typically involves obtaining a thorough medical history of the patient, a clinical assessment by a surgeon, and the utilization of laboratory tests and imaging techniques. In cases where the manifestations and signs of genitourinary and gynecological inflammatory disorders match those of AA, diagnosis can be challenging, especially in young people, elderly individuals, and females of reproductive years. Postponing an appendectomy elevates the likelihood of appendicular perforation and sepsis, subsequently leading to higher rates of hospitalization and mortality [2].

Both ultrasonography (USG) and computed tomography imaging (CT) enhance diagnostic precision [3,4]. Nevertheless, these approaches incur significant expenses and may not be readily available. Moreover, it could result in additional delays in the process of diagnosing and performing surgeries. In underdeveloped nations like India, the utilization of imaging technologies for diagnostic purposes poses a significant financial burden, and ensuring the widespread availability of radiological competence and imaging technologies in all healthcare institutions is unfeasible. Currently, multiple grading methods have been established to assist in the diagnosis of AA. The Alvarado score and modified Alvarado scoring system (MASS) are the most frequently utilized and have a range of 53-88% for specificity (SP) and 75-80% for sensitivity (SN). These scores aid in enhancing decision-making and minimizing the rate of negative appendectomies [5]. Negative appendectomies are considered undesirable outcomes as they subject patients to the risks and costs of surgery without providing any therapeutic benefit.

Negative appendectomies impose a cost on both the surgeon and patient, resulting in lost working days and decreased productivity, similar to other surgical procedures. The reported percentage of needless appendectomy typically ranges from 20% to 40% [6]. However, these scoring methods were initially established for Western people, and different investigations have demonstrated lower SN and SP when these ratings are put into effect on the Southeast Asian population. The Raja Isteri Pengiran Anak Saleha Appendicitis (RIPASA) score is a recently established diagnostic scoring system utilized in the identification of AA. Research demonstrated a markedly greater SN, SP, and diagnostic accuracy compared to the Alvarado score [7].

Kalan et al. conducted a study to evaluate the precision of the Alvarado score in the preoperative detection of AA [8]. Chong et al. conducted a prospective study on individuals with right iliac fossa (RIF) pain in a national medical center in Brunei Darussalam [7]. They found that the RIPASA scoring system, developed specifically for Southeast Asia, demonstrated high SN, SP, and diagnostic accuracy. Therefore, it is considered the most appropriate scoring system for this region. The study aimed to evaluate the association between RIPASA scores and the pathological severity of appendicitis.

## Materials and methods

An observational study was undertaken in the Department of General Surgery at MES Medical College, Perinthalmanna. The study was approved by the Ethical Committee with the letter number IEC/MES/36/2019 and proceeded for 12 months. The study comprised all patients who had been clinically diagnosed with AA, experienced RIF pain, and underwent appendectomy. Exclusion criteria for the study included individuals who declined to provide their consent alongside those with suspected appendicular mass, gynecological and urological disorders, dementia, septic shock, and signs of generalized peritonitis. A minimum estimated sample of 211 was obtained by applying the equation 4pq/E2 (p = prevalence, q = 100-p, E = allowable error) to determine the sample size with 84.3% of prevalence, and error of 5% based on an earlier study [7]. However, the final sample size was increased to 225 subjects to compensate for any attrition of the sample that was enrolled by utilizing a convenience sampling technique. The patients were evaluated using a prestructured proforma. Their RIPASA scores, which consist of 14 parameters and range from three to 16.5, were computed before the surgery. The scores were determined using the patients' demographic data (age and gender), symptoms, physical examination, and laboratory results. The estimated scores were subsequently contrasted with the histopathological results (HPR) obtained after the appendectomy. The individuals were categorized into three groups as per their RIPASA scores. The lower-score category consists of individuals who have scores ranging from 4 to 7. The intermediate-score group consists of scores ranging from 7.5 to 11.5, while the higher-score group includes values of 12 and above. These scores are associated with the HPR report, which indicates conditions such as AA, perforated appendix, appendicular abscess, or no pathology detected.

Statistical analysis

Statistical analysis was done using Statistical Product and Service Solutions (SPSS) (version 25.0; IBM SPSS Statistics for Windows, Armonk, NY). The distribution of individuals in each category was shown as percentages. The chi-square test will be employed for assessing the distribution proportions of the pathological phases of AA in the RIPASA score categories. A p-value below 0.05 was deemed to be of statistical significance.

## Results

Most of the patients were <40 years of age (78.7%). Gender distribution showed that males were the highest in the population (60.9%) (Table 1).

**Table 1:** Age- and gender-wise distribution

Variables		Frequency	Percentage
Age	<40	177	78.7
	>40	48	21.3
Gender	Males	137	60.9
	Females	88	39.1

In the study, 100% of patients had RIF pain, as it was the inclusion criteria of the study. Most patients presented after 48 hours of developing symptoms (59.6%). Anorexia is predominantly seen in patients (84.4%), rather than fever or nausea and vomiting (60% and 75.1%), respectively (Table 2).

**Table 2:** Analysis of RIPASA scoring RIPASA: Raja Isteri Pengiran Anak Saleha Appendicitis

Variable	Yes		No	
	Frequency	Percentage	Frequency	Percentage
RIF pain	225	100.0	-	-
Pain migration to RIF	165	73.3	60	26.7
Duration of symptoms (48 hours)	91	40.4	134	59.6
Fever	135	60.0	90	40.0
Anorexia	190	84.4	35	15.6
Nausea and vomiting	169	75.1	56	24.9
Guarding	74	32.9	151	67.1
RIF tenderness	225	100.0	-	-
Rebound tenderness	177	78.7	48	21.3
Rovsing's sign	72	32.0	153	68.0
Raised WBC	210	93.3	15	6.7
Negative urine analysis	205	91.1	20	8.9

Concerning the entire score, 28.4% of those assessed obtained a score higher than 12, classifying them as the high-score category. The intermediate score group comprised 60% of the subjects who scored between 7.5 and 11.5. Finally, the low-score category included 11.6% of the individuals who scored between 4 and 7 (Table 3).

**Table 3:** Categories in the RIPASA scoring system

RIPASA score	Frequency	Percentage
Low score	26	11.6
Intermediate	135	60.0
High score	64	28.4
Total	225	100.0

Out of 225 cases, 146 cases were AA (64.9%), of which 121 cases were reported in the intermediate score group (82.9%). Moreover, 27 (12%) appendicular abscess cases were noted, of which 51.9% were in the high-score group. A total of 41 (18.2%) appendicular perforation cases were observed, of which 39 were predominantly in the high-score group (95.1%), with no cases of perforation in the low-score group. The normal appendix was noted in 11 out of 225 cases in the low-score group (Table 4).

**Table 4:** Categories in the RIPASA scoring system

Histopathology report (HPR)	RIPASA score			Total n (%)	P value
	Low score n (%)	Intermediate n (%)	High score n (%)		
Acute appendicitis	14 (6.2)	121 (53.8)	11 (4.9)	146 (64.9)	0.001
Appendicular abscess	1 (0.4)	12 (5.3)	14 (6.2)	27 (12)	
Appendicular perforation	0	2 (0.9)	39 (17.3)	41 (18.2)	
No pathology found	11 (4.9)	0	0	11 (4.9)	
Total	26 (11.6)	135 (60)	64 (28.4)	225 (100)	

The association between the histopathological report and RIPASA score was found to be statistically highly significant (p=0.001). In the low-score group, there were 14 cases of appendicitis (53.8%), one case of appendicular abscess (3.8%), a total of 11 cases without pathology observed (42.3%), and no reported instances of appendicular perforation. In the intermediate-score category, there were 121 cases of appendicitis (89.6%), 12 cases of appendicular abscess (8.9%), 2 cases of appendicular perforation (1.5%), and no reported cases in the non-pathology category. Among the high-score category, there were 11 cases of appendicitis (17.2%), 14 cases of appendicular abscess (21.9%), 39 cases of appendicular perforation (60.9%), and no reported instances of negative appendectomy.

## Discussion

Since the inception of test scoring systems, numerous studies have been conducted to identify the optimal SN, SP, and diagnostically precise clinical grade for diagnosing AA. Alvarado score has gained significant recognition and has been extensively examined as a scoring system for AA [9]. Its modified version (MASS) has also been widely utilized [10]. Given the widespread usage and popularity of this scoring framework, we aim to examine the recently developed method of evaluation (RIPASA) and how it correlates with the pathological severity of appendicitis. Our study findings indicate that most patients were men, accounting for 60.9% of the total. Furthermore, a significant proportion of patients, specifically 78.7%, belonged to the younger age group of under 40 years old. Most individuals sought medical attention 48 hours following the onset of symptoms (59.6%). Anorexia is more prevalent in patients (84.4%) compared to fever or nausea and vomiting (60% and 75.1%, respectively).

Among those in the group with RIPASA values ranging from 4 to 7 (indicating a low score), 6.2% were diagnosed with AA, 0.4% had an appendicular abscess, and 4.9% did not have any detectable pathology. Within the cohort of participants with RIPASA scores ranging from 7.5 to 11.5, the prevailing condition was appendicitis, affecting more than half of individuals (53.8%), while appendicular abscess was observed in 5.3% of cases. Within the high-score category, 17.3% of individuals experienced appendicular perforation, while 6.2% and 4.9% experienced appendicular abscess and AA, respectively. No instances of appendicular perforation were observed in the low-score group. However, as the RIPASA levels increased, the clinical conditions of these individuals worsened. Similarly, within the intermediate and high-score categories, there were no instances where no pathology was detected.

The diagnosis of AA can be difficult in children, teenagers, elderly individuals, and females of reproductive age because of the wide range of potential diseases and the presence of atypical clinical signs [11]. Under such instances, the utilization of sophisticated radiological assessments including CT may be required [12]. Nevertheless, there are instances where the identification of AA can solely rely on the visual assessment of the appendix tissue during surgery and the subsequent analysis of the surgically excised appendectomy tissue using histological techniques [13]. The Alvarado scoring and modified Alvarado scores, originally developed in the Western context, showed low levels of SN and SP when employed in Asia and the Middle East [14]. Khan et al. found that the Alvarado scoring method demonstrated an SP of 23% and SN of 59% when employed in an Asian population [15]. Al-Hashemy et al. demonstrated that the MASS technique had an SP of 80% and an SN of 53.88% when applied to a Middle Eastern population [16]. To address this issue, the RIPASA score was established at a medical facility in Darussalam [17]. The method is easy to use, and secure, with enhanced diagnostic precision, particularly for those of Asian descent residing in remote areas with inadequate access to or financial means for radiological diagnostic equipment. The scoring method was explicitly customized for Asians owing to the inadequate suitability of the Alvarado score and MASS for South Asian populations in general, as seen by ethnic disparities [14-16].

Groundbreaking research conducted by Chong et al. [7] was a retrospective analysis that utilized data from 312 individuals who underwent an emergency appendectomy as their primary surgical treatment. A group of surgeons at the medical center together determined 14 evaluation criteria, along with one extra criterion tailored to their particular population. The RIPASA score is currently determined by these 14 criteria. The study utilized the data to construct a receiver operating curve and determine an ideal threshold value of 7.5 that yielded an SN of 88% and SP of 67%, as well as a positive predictive value (PPV) of 93% and a negative predictive value (NPV) of 53%. According to their study, the newly developed RIPASA score demonstrated superior diagnostic accuracy for AA in a South Asian population as opposed to both the Alvarado score and the MASS. The researchers behind the study acknowledged that although the investigation was conducted on a specific group of patients in their local area, the findings can be relevant to other groups in Southeast Asia and the Middle East.

Research indicates that doing redundant CT scans on people who have early low-grade appendicitis can result in superfluous appendectomies. However, it has been found that these cases can be treated well with antibiotics, leading to spontaneous resolution. Consequently, subjecting such patients to surgical procedures exposes them to avoidable risks [12,18,19]. Subsequently, another study designed by Chong et al. [18] was carried out one year afterward, with an additional 192 individuals. On this occasion, they achieved a RIPASA score of 98%, 81.3%, 85.3%, 97.4%, and 91.8%, respectively, for SN, SP, PPV, NPV, and diagnostic accuracy, as opposed to 68.3%, 87.9%, 86.3%, 71.4%, and 86.5%, respectively, for the MASS. The researchers determined that the inclusion of extra parameters in the RIPASA score enhanced its flexibility and adaptability across various geographical regions.

However, in an emergency, the healthcare professional on duty can promptly determine which individuals with RIF pain should be referred to the surgical team for admission using a RIPASA score of greater than or equal to 7.5. Individuals with a RIPASA score of <7.0 may either remain under observation in the hospital ward or be discharged with instructions to monitor their condition. Therefore, the RIPASA Score is presently a superior diagnostic scoring technique for AA compared to the Alvarado rating. The former demonstrates considerably greater SN, SP, NPV, and diagnostic precision, especially in the context of the Indian population [20].

The omission of those with comorbidity and other illnesses may restrict the relevance of the results to a broader patient population. Although there are constraints, the study offers vital insights into the application of the RIPASA score in diagnosing AA. Even while additional local and regional investigations have also been conducted on a comparable proportion of patients, a sample size of just 225 individuals may not be adequate to generalize the results to a larger population [17,21]. In a study conducted in India, 206 patients were examined. The results indicated that the RIPASA score had a greater degree of SP (90.5%) and SN (96.2%) compared to the MASS, whereas the MASS had an SN of 58.9% and an SP of 85.7% [2]. Another investigation conducted in India demonstrated that the MASS had an SN of 64.44% and an SP of 58.82%, whereas the RIPASA score had an SN of 87.78% and an SP of 76.47% [22]. Research conducted in Jordan [23], Kuwait [21], Iran [24], and Turkey [5], together with a multicenter, cross-border study involving Saudi Arabia and Egypt [25], have confirmed the findings of the initial study in the Middle Eastern population. Malik et al. conducted one of the initial assessments of the RIPASA scoring in a Western population in Ireland, following the observation of favorable outcomes in several Eastern investigations [26]. In their retrospective analysis of 208 patients, the researchers achieved an SN of 85.93%, an SP of 69.86%, and a diagnostic accuracy of 80.01%. Greek scholars presented a meta-analysis of 12 studies that contrasted the RIPASA scores with the Alvarado score. The SN of the RIPASA and Alvarado scores were 94% and 69%, respectively, while their SP was 55% and 77%. The RIPASA score had a computed diagnostic precision of 94.3%, while the Alvarado score showed a diagnostic accuracy of 79.4% [9]. A recent retrospective investigation indicates that appendectomy is unlikely to have an impact on the occurrence of intra-abdominal abscesses in both uncomplicated and complicated cases of appendicitis. Nevertheless, laparoscopic appendectomy offers the benefits associated with laparoscopic techniques, including reduced hospitalization time and faster resumption of regular daily tasks. Consequently, it is advisable to prioritize laparoscopic appendectomy for cases of AA [27]. Higher scores are attained in the RIPASA scoring method when the degree of inflammation in the appendix tissue increases. This has an impact on the clinical and laboratory results. Considering the possibility of encountering a more complex case of appendicitis during the procedure, the surgical team will be better equipped to make the choice on a procedure and take the appropriate precautions when dealing with an RIPASA high-scoring patient.

The study limitations include are as follows. First, the study was conducted as a single-center investigation at the Department of General Surgery at MES Medical College Perinthalmanna. This single-center design may limit the generalizability of the findings to broader populations and different healthcare settings. Second, the sample size of the study was relatively small, comprising a convenience sample of 225 individuals. A larger sample size would have provided greater statistical power and increased the reliability of the study findings. Furthermore, the lack of blinding among clinicians assessing RIPASA scores and interpreting histopathological results may have introduced bias into the study. Blinding could minimize the risk of subjective interpretation and enhance the objectivity of the diagnostic correlation between RIPASA scores and histopathological findings.

## Conclusions

This study showed that the RIPASA evaluation method possesses exceptional diagnostic accuracy in identifying AA. The scoring technique is a simple, cost-effective, nonintrusive consistent, and secure diagnostic method that does not require additional expenses or challenges. It is simpler to adhere to at peripheral institutions with limited reserves for backup. When used correctly and without prejudice, it can greatly assist surgeons at peripheral clinics when diagnosing those with probable appendicitis. Implementing this scoring method enhances diagnostic precision, leading to a decrease in the rate of negative appendectomies and complications.
